# Plan for the next step after a failed transfer cycle: “Doctor, what are we going to do differently?”

**DOI:** 10.1016/j.xfre.2026.02.003

**Published:** 2026-02-18

**Authors:** Richard J. Paulson

**Affiliations:** USC-HRC Fertility, Division of Reproductive Endocrinology and Infertility, Department of Obstetrics and Gynecology, Keck School of Medicine, University of Southern California, Los Angeles, California

I hate failed transfer cycles. I hate failure in general, but a failed transfer cycle doesn’t just mean that I failed, but more specifically, I failed someone else. Someone came to me for help, decided to put their trust in me, I did my best—but my best wasn’t good enough, because the cycle failed. The patient is unhappy at best, devastated at worst, and I feel terrible. After 40 years of transferring embryos, I’m certainly accustomed to negative outcomes, but they remain frustrating and very unpleasant.

The ensuing patient follow-up visit results in a familiar discussion. What happened? The embryologist said the embryo looked healthy, the doctor said that the uterine lining was thick enough, and the transfer went smoothly. We all saw the catheter on ultrasound, entering the uterus, placing the embryo in the right location, the patient took all the prescribed medications, so how come the cycle failed? More importantly, when planning the next embryo transfer, “Doctor, what are we going to do differently so that we don’t have the same bad outcome?”

Every fertility specialist faces this question. Even if the cycle went according to plan, and everything was done correctly, each clinician feels the pressure to do additional testing, offer an unproven—even controversial—treatment, just to satisfy the patient’s request that “something different” be done. I know this to be true, because colleagues and former fellows call me regularly to discuss how best to navigate this situation. This is why so many useless diagnostic and therapeutic interventions continue to thrive, and even proliferate.

My suggestion is to review the cycle, step by step, and confirm that there was nothing that needed improvement. Since I like to think of embryo implantation in mechanical terms, I approach this problem from an engineering perspective, complete with a mathematical formula that I write out for each patient (1). Embryo implantation (EI) is described as consisting of three general components: embryo quality (EQ), endometrial receptivity (ER) and transfer efficiency (TE). In mathematical terms,

EI=EQxERxTE

Working backward, I explain that “transfer efficiency” signifies the importance of the embryo transfer process; if the embryo is somehow not delivered into the endometrial cavity, or if the uterus contracts and expels the transferred embryo, implantation will not occur. In the past, embryo transfers were often more challenging. Patients were advised to have an empty bladder, rigid transfer catheters were guided by surgical “feel” and a tenaculum might have been used to straighten the cervical canal. With increasing experience and data, we gradually came to use soft catheters to minimize uterine cramping and ultrasound guidance to minimize trauma to the endometrium and ensure that the embryo was placed in the right location. As a consequence, TE is now very good and is an uncommon cause of implantation failure.

The middle term, “endometrial receptivity,” represents the ability of the uterine lining to accept the implantation. It is maximized by ensuring that the cavity is normal and by providing adequate estrogen priming so that progesterone receptors are sufficient to allow the action of progesterone to open the window of implantation. We document the adequacy of estrogen priming by measuring the endometrial thickness. Although this measurement is far from perfect, it is useful and noninvasive. Finally, the embryo should be transferred after the correct duration of progesterone administration, between approximately 96 and 120 hours (2). It is important to stress that no other intervention has been definitively shown to improve receptivity. Therefore, if we documented a normal cavity, adequate endometrial thickness, and the correct duration of progesterone prior to the transfer, the endometrium has been optimized and is also an unlikely cause of implantation failure.

We are left with “embryo quality” as the final—and most important—factor in implantation. We know that available methods of measuring embryo quality are not perfect. Even embryos with perfect marks for appearance, growth and karyotype do not have a 100% chance of implantation. Under the best of circumstances, at the present time, we may achieve implantation rates of about 70% (3). In most cases, depending on the clinical setting, implantation rates are substantially lower. In the language of science, we can say that embryo quality is the rate-limiting step of embryo implantation.

It is worth noting that whether those implantation rates are 30% or 70%, studies evaluating successive embryo transfer cycles have consistently shown no decrease in implantation rates with serial transfers in the same patients (4, 5). If there were an undiagnosed cause of implantation failure, serial implantation rates should logically decrease in subsequent cycles. This is because those with the putative implantation problem would become more concentrated in the remaining not-yet-pregnant population. However, that is not the case (4, 5), and we should reassure our patients that, “rate of true recurrent implantation failure is low (5).”

What are we going to do differently? If everything was done correctly in the previous cycle, then nothing needs to be done differently. We have good data to suggest that the next cycle will have the same chance of success as the previous cycle (4, 5). Unproven diagnostic testing and treatments only waste time and resources. The best course of action is to perform another transfer cycle.

What are we going to do differently?

We are going to transfer a different embryo.

### Declaration of Generative AI and AI-Assisted Technologies in the Writing Process

During the preparation of this work the author used ChatGPT to ensure the accuracy of grammar and spelling. After using this service, the author reviewed and edited the content as needed and takes full responsibility for the content of the published article.

## Declaration of Interests

R.J.P. has nothing to disclose.
